# Alendronate versus Raloxifene for Postmenopausal Women: A Meta-Analysis of Seven Head-to-Head Randomized Controlled Trials

**DOI:** 10.1155/2014/796510

**Published:** 2014-01-05

**Authors:** Tiao Lin, Shi-Gui Yan, Xun-Zi Cai, Zhi-Min Ying, Fu-Zhen Yuan, Xi Zuo

**Affiliations:** ^1^Department of Orthopaedic Surgery, Second Affiliated Hospital, School of Medicine, Zhejiang University, Hangzhou, Zhejiang 310009, China; ^2^Department of Orthopaedic Surgery, Perelman School of Medicine, University of Pennsylvania, Philadelphia, PA 19014, USA; ^3^Department of Epidemiology and Biostatistics, School of Public Health, Drexel University, Philadelphia, PA 19102, USA

## Abstract

*Purpose*. The aim of this study was to directly compare the efficacy and the safety of the two agents for postmenopausal women. *Methods/Principal Findings*. Electronic databases were searched for relevant articles that met our predefined inclusion criteria. Seven randomized controlled trials (RCTs) involving 4054 women were identified and included. Although Aln was more effective than Rlx in increasing bone mineral density (BMD), no statistical differences were observed in reducing the risk of neither vertebral fractures (*P* = 0.45) nor nonvertebral fractures (*P* = 0.87) up to two-year followup. Aln reduced the risk of vasomotor (*P* = 0.006) but increased the risk of diarrhea compared to Rlx (*P* = 0.01). Our subgroup analysis further indicated the difference between Aln and Rlx in fracture risk and was not materially altered by the administration pattern, the age. The weekly strategy of Aln would further reduce the upper gastrointestinal (GI) disorders and might gain more bone mass increment at lumbar spine compared to its daily treatment. *Conclusion*. There was no evidence of difference of fracture risk reduction between Aln and Rlx. In addition, age did not obviously influence their relative antifracture efficacy. For Aln the weekly strategy would further reduce the upper GI disorders and gain more bone mass increment compared to the daily treatment. During clinical decision making, the patients' adherence and the related side-effects associated with both drugs should also be taken into account.

## 1. Introduction

Osteoporotic fracture is a world-wide concern in the current aged society. It is estimated that annually there are 180,000 people encountering osteoporosis-related fractures in England and Wales. Postmenopausal women with bone loss were considered at high risk of bone fractures, which greatly impaired their life quality and led to mortality [1]. An appropriate and timely management for preventing osteoporotic fracture is extremely important. At present, antiresorptive agents are still the major treatments. Besides the novel Denosumab, which is a human monoclonal antibody of receptor activator of NF-*κ*B ligand (RANKL) and potently suppresses osteoclastic bone resorption, alendronate (Aln), the most widely prescribed bisphosphonates, and raloxifene (Rlx), the only Food and Drug Administration approved selective estrogen receptor modulators (SERMs), are the most evident antiresorptive agents for prevention and treatment of postmenopausal osteoporosis [2, 3].

For deciding the therapeutic strategy, it is highly imperative to know an estimate of the difference in fracture risk reduction between Aln and Rlx [4, 5]. Although both therapies have established efficacy from randomized controlled trials (RCTs), Rlx was suggested to be less effective compared to Aln, mainly in preventing nonvertebral fractures [3, 6–8] and was therefore not recommended as a first-line treatment option for this population or considered as an alternative for young women with lower nonvertebral risk [3, 9].

However, so far the efficacy inferiority of Rlx under Aln for postmenopausal women, especially the most relevant outcome fractures prevention, was still inconclusive. (a) The evidence was mainly derived from the indirect comparison with placebo, of which the inherent defects should be respected [10, 11]. With the statistical methods of indirect comparison such as the Bayesian method and the network meta-analysis [6, 12, 13], there were significant inconsistencies in patient characteristics across the included studies. In particular, the population of the previous systematic reviews and meta-analyses were consisted of elderly osteoporotic men and patients with glucocorticosteroid-induced osteoporosis apart from postmenopausal women [6, 8, 12]. In addition, the adverse effects (AEs) of two agents, which would highly provide reference during clinical decision making, were not thoroughly compared in previous meta-analyses. (b) Recently, a large-scaled retrospective study conducted by Foster et al. [14] emphasized that after adherent treatment there was a similar risk reduction for the both drugs in both vertebral and nonvertebral fractures of women up to 8 years, which was inconsistent with the previous prospective evidence [6, 12, 13]. (c) There were emerging head-to-head RCTs to evaluate the comparative effectiveness of the two agents, the results of which were mainly limited by the small sample size but those available comparative data should be well summarized and taken into consideration [15–21].

Taken together, this meta-analysis with all the available 7 head-to-head RCTs involving 4054 participants was conducted to summarize the comparative efficacy of bone mass increment and fracture prevention between Aln and Rlx for postmenopausal women [15–21]. Their safety profiles were also reviewed in a head-to-head comparative manner. Besides, we aimed to evaluate clinically-related and design-related factors which might contribute to the difference in efficacy and AEs.

## 2. Methods

### 2.1. Literature Search

Electronic databases (PubMed, Medline, EMBASE, Clinical Trial Registry and the Cochrane Data Base of Systematic Reviews, and the Cochrane Central Register of Controlled Trials) were searched without limit by two independent investigators (Lin and Ying), which were last updated on October, 2013. The search used terms and Boolean operators as follows: “(alendronate OR bisphosphonate) AND (raloxifene OR selective estrogen receptor modulators) AND postmenopausal women AND (osteoporosis OR fracture).” Reference lists of all the selected articles were hand-searched for any additional trials.

### 2.2. Identification of Eligible Studies

The trials were reviewed in which (a) the target population were consisted of postmenopausal women with low bone mass, (b) the interventions at least included both Aln and Rlx therapies, (c) the outcomes at least comprised one of the following assessments: fracture incidence, BMD, or safety profile, and (d) the trials were randomized controlled trials (RCTs). The trials were excluded if (a) patients had a prior history of metastatic bone disease, (b) phase-I or observational studies, case reports, and reviews, and (c) the same RCTs were reanalyzed. Disagreements were resolved through discussions.

### 2.3. Assessment of Study Quality

Two reviewers (Lin and Ying) independently assessed the study validity with Cochrane Collaboration's tool for assessing the risk of bias, which addresses six specific domains such as sequence generation, allocation concealment, blinding, incomplete outcome data, and selective outcome reporting. Whether the included trials were similar in baseline, adopting similar cointerventions, and applying intention-to-treat (ITT) analysis was also evaluated. Disagreement was evaluated by means of kappa (*κ*) test and resolved by discussion [22].

### 2.4. Data Abstraction, Conversion, and Analysis

For each eligible trial, two of us (Lin and Ying) independently extracted the relevant data and checked the accuracy. In particular, we abstracted study design, sample size, demographic data (age, body mass index, and baseline BMD), intervention protocol, duration of the trial, loss to followup, trial outcomes (fracture incidence, BMD, and incidence of adverse events), and industrial funding. We contacted the first or the corresponding author of each eligible trial to verify the accuracy of the data abstraction as well as our methodological assessment.

The overall incidences of vertebral or nonvertebral fractures (hip, upper leg, lower leg, pelvis, hummers, wrist/forearm, clavicle/rib, and other) in the two groups were our primary outcome. We also evaluated the BMD percentage changes from the baseline at lumbar spine (LS), femoral neck (FN), and total hip (TH) in both groups. BMD was measured by dual-energy X-ray absorptiometry (DXA). The safety profile comprised the reported discontinuations due to AEs, AEs probably related to Aln (upper gastrointestinal disorders (GI) and diarrhea), and AEs probably related to Rlx (vasomotor events and venous thrombosis).

We took fracture risk reduction, LS BMD, and risk of upper gastrointestinal (GI) disorders at the end of follow-up as our main meta-analysis on basis of their sufficient trials for subgroup analysis.

We preferentially used the ITT data from the trials whenever possible. If the data were not reported in the original article, we extrapolated them from the accompanying graphs. To maximize data availability, we applied percentage change data for BMD and serum lipid outcome. If percentage change data were unavailable in BMD outcome, we imputed the percentage change data using (endpoint data, baseline data) divided by baseline data then multiplying 100 times. For the missing standard errors (SEs) of BMD data, the maximum SEs extracted from Muscoso et al. [18] were conservatively chosen for all BMD percentage change SEs. The sensitivity analysis was performed through omitting trials with imputed SEs to assess the variation in overall effect [22].

The fracture incidence and the safety profile outcomes were presented as risk ratio (RR) with 95% confidence intervals (CI) and combined using the Mantel-Haenszel method. BMD were pooled with the inverse variance method and presented as weighted mean differences (WMD) and 95% CI. We calculated the statistical heterogeneity using a Chi-squared (*χ* ^2^) test with the significance at 0.1. We also assessed the inconsistency *I* ^2^ to describe the percentage of the variability in effect estimates due to the heterogeneity. We considered a value greater than 50% as the substantial heterogeneity. Fixed effects model would be applied if there were no statistical heterogeneity among the studies; otherwise, we used the random effects model. If substantial heterogeneities across studies (*I* ^2^ > 50%) were detected in the index five main meta-analysis, we performed post hoc sensitivity analysis by omitting the outlier studies to determine the sources of Cochran's heterogeneity [22]. The outliers were detected as the studies with confidence interval of the estimated effect size were not well overlapping with the pooled overall effect size [23].

The subgroup analyses in the main meta-analysis were performed by baseline characteristics of the studies: patterns of treatments in Aln groups (daily or weekly), mean age of participants (>65 or ≦65), methodological quality, sample size (≧400 or <400), and industrial funding. BMI of participants and dose of agents could not be analyzed in subgroup analysis due to the difficulties in determining cut-off values. To determine the influence of outlier studies, after omitting the two detected outliers, the pooled-analysis and the subgroup analyses were repeated in the main analysis with statistical heterogeneities. Results of subgroup analysis were presented only if each subgroup comprised at least two trials.

To comprehensively identify the clinical-related modifiers, metaregression with covariates (age, BMI of participants, patterns of Aln administration) were carried out in the fracture (vertebral fracture analysis was not performed as only 3 trials included) and GI disorder analysis.

To evaluate the publication bias, we used Begg's test and Egger's test with trials from fracture outcomes analysis, including 6 trials in total fractures, 3 trials in vertebral fractures, and 4 trials in nonvertebral fractures [22].

Meta-analysis was conducted using Review Manager 5.1 software. Metaregression analysis, Begg's test, and Egger's test were performed through STATA 11.0 (Stata Corp, College Station, TX, USA). The criteria of the Grading of Recommendations Assessment, Development, and Evaluation (GRADE) were used to evaluate the quality of evidence by each outcome [24].

## 3. Results

### 3.1. Study Identification

**Figure 1:**
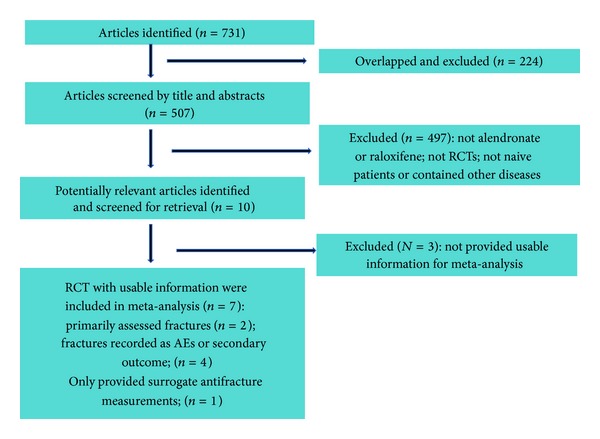
Flow chart for the meta-analysis.

Literature search initially yielded 731 relevant articles; of which 224 overlapped publications were excluded. From the remaining 507 articles, 497 were excluded since they did not fulfill the selection criteria based on their titles and abstracts. After full-text checking of the rest of 10 RCTs [15–21, 25–27], 3 RCTs were excluded as their outcomes did not meet the inclusion criteria [25–27]. Finally, 7 studies with usable information were included in our meta-analysis [15–21] (Figure 1). The weighted kappa for the agreement on eligibility between reviewers was 0.81 (95% CI: 0.72–0.90).

### 3.2. Study Characteristics

The characteristics of the included 7 trials were shown in Table 1 [15–21]. Four of the trials were double blinded and placebo-used, multicenter RCTs [16, 17, 19, 20]. There were two studies designed with fractures as endpoint in a two-year followup [18, 19]. Other five studies [15–17, 20, 21] with one-year followup used BMD as surrogates for antifracture assessment, and the fractures was reported as AEs [16, 17, 20] or secondary outcomes [15, 18]. Four studies only comprised Aln and Rlx treatments [15, 17, 19, 20]. The other three studies contained combined treatment [16, 21] or other therapies as well [18]. Only one trial was considered to have substantial loss to follow-up (more than 20%) [21], while the rates were acceptable among the other studies (range, 1.7% to 19.7%). Two studies were funded by Aln Company (Merck & Co.) [17, 20] and two were funded by Rlx Company (Eli Lilly & Co.) [16, 19], while the left three did not involve any industrial funding [15, 18, 21]. In terms of the patterns of administrations in Aln groups, four studies treated women once daily [15, 16, 18, 19], while the other three adopted once weekly strategy [17, 20, 21]. Patients in the both groups took calcium and vitamin D as supplementations equally in all eligible studies.

### 3.3. Study Quality

The methodological quality was evaluated independently by two reviewers (Lin and Ying) with Cochrane Collaboration's tool for assessing the risk of bias and showed in Table 2 [22]. Four trials [6, 7, 9, 10] described explicit adequately randomization, concealment of allocation assignment, proper blinding, and applying intention to treat analysis, which were low risk of bias [16, 17, 19, 20], while the other three trials with inexplicit randomization and inadequate blinding were considered moderate risk of bias [15, 18, 21]. The weighted kappa for the agreement on the trial quality between reviewers was 0.84 95% CI: (0.75–0.93).

### 3.4. Effect of Interventions

**Figure 2:**
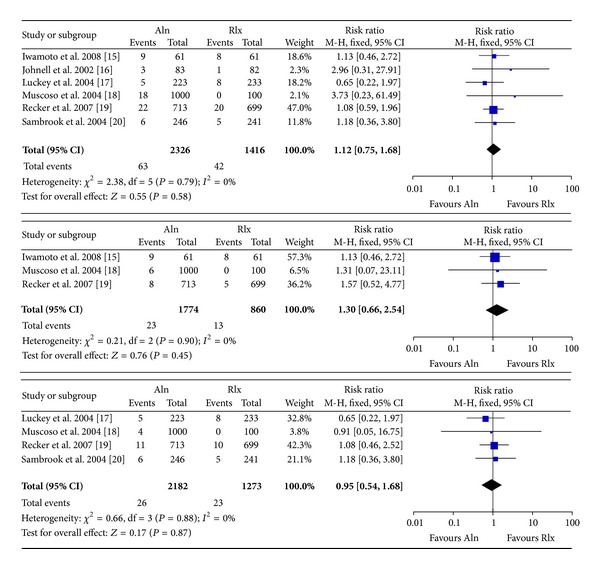
Relative risk of total fractures, vertebral fractures, and nonvertebral fractures for postmenopausal women between the alendronate group and the raloxifene group. NV: nonvertebral.

No differences in total, vertebral or nonvertebral fracture incidences were demonstrated between the Aln groups and Rlx groups. (Total: 6 studies, fixed-effects RR (95% CI): 1.19 (0.75, 1.68), *P* = 0.58, *I* ^2^ = 0%; vertebral: 3 studies, fixed-effects RR (95% CI): 1.30 (0.66 to 2.54), *P* = 0.45, and *I* ^2^ = 0%; nonvertebral: 4 studies, fixed-effects RR (95% CI): 0.95 (0.54, 1.68), *P* = 0.87, and *I* ^2^ = 0%, Figure 2). Our meta-analysis indicated moderate quality evidence of equivalent efficacies between the two medications in fractures prevention (Table 3).

All of the included studies reported BMD data measured by DXA at least at one skeleton site. Both Aln and Rlx increased BMD significantly at LS, FN, and TH after 6, 12, and 24 months related to the baseline. Aln obtained bone mass increment to a greater extent than Rlx (Table 4), and the differences were widening as the treatment continued. The evidence quality was moderate (Table 3).

**Figure 3:**
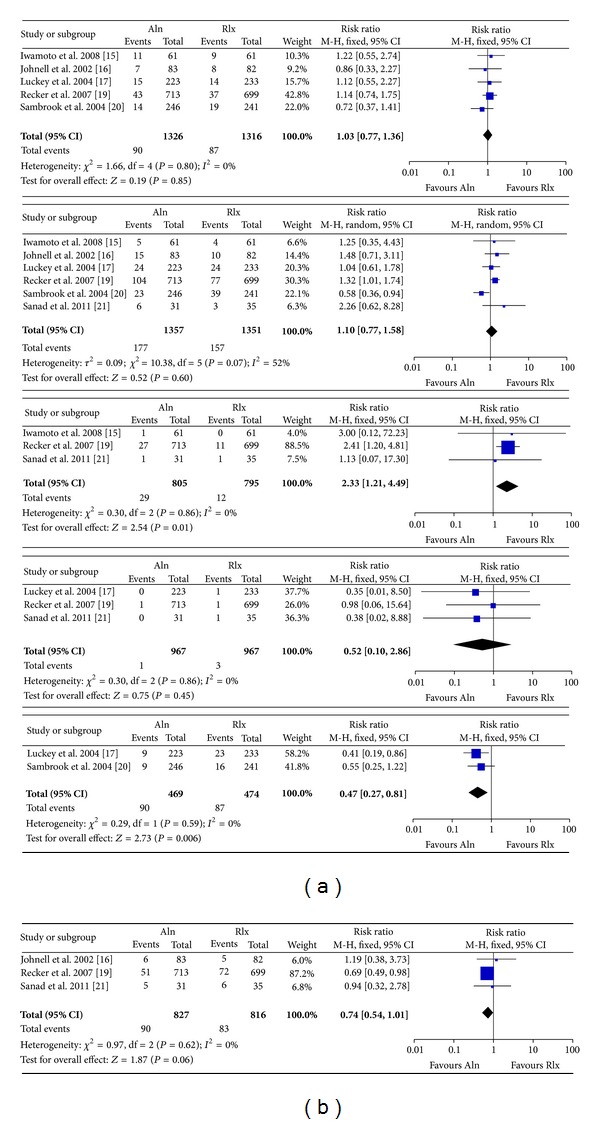
Relative risk of discontinuations due to adverse events, upper gastrointestinal disorders related to treatment, diarrhea events, venous thrombosis events, vasomotor events, and vasodilatation events for postmenopausal women assigned to alendronate compared with raloxifene.

Both Aln and Rlx were well tolerated, no fatal AEs related to treatment were reported. It was similar in drop out due to AEs, upper GI disorders, venous thrombosis, and vasodilatation in the both groups: (Aln versus Rlx: drop out due to AEs: 5 studies, RR: 1.03 (0.77 to 1.36), *P* = 0.85, and *I* ^2^ = 0%; upper GI disorders: 6 studies, RR: 1.10 (0.77 to 1.58), *P* = 0.60, and *I* ^2^ = 52%; venous thrombosis: 3 studies, RR: 0.52 (0.10 to 2.86), *P* = 0.45, and *I* ^2^ = 0%; vasodilatation: 3 studies, RR: 0.74 (0.54 to 1.01), *P* = 0.06, and *I* ^2^ = 0%, Figure 3). And the evidence quality for the differences among those AEs risks was moderate to high with the exception for Aln increase greater risks of upper GI disorders than Rlx, which was supported by low quality evidence (the quality of evidence turned out to be high if excluding the outlier study [20]) (Table 3).

Moderate to high quality evidence showed that Aln would increase 133% risks of diarrhea while avoid 57% risks of vasomotor events compared to Rlx (Aln versus Rlx: diarrhea: 3 studies, RR: 2.33 (1.21 to 4.49), *P* = 0.01, and *I* ^2^ = 0%; vasomotor: 2 studies, RR: 0.47 (0.27 to 0.81), *P* = 0.006, and *I* ^2^ = 0%, Figure 3).

### 3.5. Heterogeneity and Outlier

Of the main meta-analysis, substantial heterogeneities were detected in outcomes of LS BMD (at 12 months: *P* < 0.01, *I* ^2^ = 95%) and upper GI disorders (*P* = 0.60, *I* ^2^ = 52%). In LS BMD comparison (at 12 months), Iwamoto' study was found as an outlier [15]. After omitting this study, the results showed insignificant heterogeneities across studies (*P* = 0.19, *I* ^2^ = 35%) and the estimate effect size (WMD) in LS BMD was only reduced from 2.92 (95% CI: 2.23 to 3.62) to 2.37 (2.17 to 2.58). Sambrook's research turned out to be an outlier in the GI disorders [20]. The heterogeneities in GI disorders were simultaneously reduced to be minimal (*P* = 0.83, *I* ^2^ = 0%) after excluding this study while the differences between Aln and Rlx in risks of GI disorders turned out to be statistical (Aln versus Rlx: RR: 1.30 (1.04, 1.63), *P* = 0.02). Since the two outlier studies were identified, the subgroup analysis were repeated after excludion of them in LS BMD at 12 months and GI disorders respectively.

### 3.6. Sensitivity Analysis and Subgroup Analysis

The overall results of main meta-analysis were not significantly altered by omitting trials with imputed SEs. Our subgroup analysis suggested that patterns of administrations in Aln groups, participants' age, methodological quality, sample size, or industrial funding of included studies were not associated with the overall effect size of the differences in fracture reduction. The outlier studies did not alter the results of the subgroup analysis in incidences of GI disorders [15, 20].

The higher risk of Aln in upper GI disorders compared to Rlx was detected in the subgroups containing studies with daily administrated Aln (Aln versus Rlx: RR: 1.34 (1.04, 1.72), *P* = 0.02) and with participants over 65 years old (Aln versus Rlx: RR: 1.32 (1.01, 1.73), *P* = 0.04), which remained unchanged either including or excluding Sambrook et al. [20]. Notwithstanding, after excluding Iwamoto's study [15], the studies involving weekly treated Aln groups contributed to a greater difference in LS bone gain between Aln and Rlx groups compared to those which adopted daily strategies in Aln groups (weekly versus daily: WMD difference: 0.36, *P* = 0.01), while the difference was not statistical (*P* = 0.26) under the presence of Iwamoto's study [15] (Table 5).

### 3.7. Meta-Regression Analysis

Women's age, BMI, and pattern of Aln administration have no obvious impacts on the results of fracture (total and nonvertebral fractures) analysis in our metaregression analysis. Though it was insignificant, a widening difference was observed that Aln had more upper GI disorders over Rlx when women's propensity to adopt daily Aln administration or participants' age increased (Supplementary file 1 in the Supplementary Material available online at http://dx.doi.org/10.1155/2013/796510).

### 3.8. Publication Bias

We found no evidence for publication bias both in vertebral fractures (3 trials) and nonvertebral fractures (4 trials), according to both Begg's test and Egger's test [28]. Although the Begg's test funnel plot indicated a potential absence of small size studies which favored Rlx groups in total fractures (6 trials), a trim and fill analysis suggested there were probably 2 missed small trials and the effect size (RR) would be more close to 1 by including them (Supplementary file 2).

## 4. Discussion

Our meta-analysis suggested no superiority of Aln over Rlx in reducing the risk of both vertebral fractures and nonvertebral fractures within a followup of 12–24 months. Aln was more effective in increasing BMD than Rlx. Aln reduced the risk of vasomotor by 57% but increased the risk of diarrhea by 133% compared to Rlx. Our subgroup analysis further indicated that the difference between Aln and Rlx in fracture reduction was not materially altered by administration pattern, age, methodological quality, sample size, or industrial funding. The weekly strategy of Aln would further reduce the upper GI disorders and might gain more bone mass increment compared to its daily treatment.

### 4.1. Strength and Evidence Quality

Our meta-analysis was the first to exclusively comprise head-to-head RCTs, target postmenopausal women and comprehensively evaluate the fracture risk, BMD, and the adverse effects. The previous systematic reviews and the network meta-analyses had indirectly compared the two agents within their multiple agents [2, 6, 8]. Based on the data of the individual agent compared with the placebo, however, their results had poor consistency and great bias due to the variation in the baseline characteristics of participants and the administration pattern of drugs among the trials [10, 11]. The validity of our findings was further strengthened by strictly following “Cochrane Handbook for systematic Reviews of Interventions 5.0.2” [22]. In particular, we developed the clear criteria of inclusion and exclusion, thoroughly assessed the methodological quality of the included studies, and embarked on the quantitative analysis. Identification of the outlier studies and the sensitivity analysis was to sort out the source of heterogeneity in the present analysis, with the purpose of verifying the results. We also performed the subgroup analysis to comprehensively evaluate the multiple factors potentially influencing the comparative effect. Finally, we used the GRADE system to rigidly assess the quality of evidence, which we aimed to recommend for both agents [24]. Generally, our GRADE analysis showed the evidence of moderate to high quality in most endpoints, which was higher than the previous pooled analyses [6, 12] (Table 3).

### 4.2. Limitations

(a) The combined sample size in current meta-analysis was still limited. However, a large-scale comparative RCT trying to achieve significant fracture prevention difference of the two agents was destined to be infeasible and unnecessary, as the sample size would be unfortunately and unbelievably huge (given the risk fracture of Aln and Rlx in the present analysis was 2.71% and 2.96%, it would need over 100,000 patients to confirm the theoretical difference of 0.25%) [29]. In fact, a well-conducted meta-analysis would always economically and adequately reflect the results of the large-scaled RCT [30]. The present analysis involving all available comparative RCTs with moderate to high quality evidence on the two therapies for postmenopausal women may provide important information for health care providers to supplement the clinical trial evidence. (b) The heterogeneity was detected in the outcome of LS BMD and GI disorder. The outlier was identified as Iwamoto et al. [15] in LS BMD and Sambrook et al. [20] in GI disorders. The age of each study (Iwamoto et al.: 69.4, Sambrook et al.: 61.6) which differed from that of the other trials (62.1~67.5) might be the contributor (Table 1). (c) Three studies, lacking adequate randomization, blinding, and concealment of allocation, were considered as the moderate quality [15, 18, 21]. One of them also encountered the loss to followup of certain degree [21]. However, our subgroup analysis suggested the conclusions were not overall influenced by the trial quality. (d) Four studies [16, 17, 19, 20] were sponsored by the pharmaceutical companies related to the either agent. Although the bias of the selective reporting should be considered, the industrial funding was found not to alter the overall results.

### 4.3. Interpretation and Clinical Implications

Aln was well proved to be more potent than Rlx in inhibition of resorption [31]. Aln could tightly bound to trabecular surfaces where osteoclasts attached and then disrupted their function after its ingestion [32]. As for Rlx, it bound to the raloxifene-estrogen receptor and activated a specific sequence of DNA known as the Raloxifene Responding Element. The subsequent increasing expression of specific cell proteins, which acted as estrogen agonist, resulted in osteoclast suppression [33]. Briefly, the more significant efficacy of Aln over Rlx in BMD increment is probably due to their different pathways of antiremodeling effect [28, 34, 35].

However, a discrepancy between the statistical difference in bone mass increment and their similar efficacy in vertebral and nonvertebral fracture prevention in the current analysis ought to be cautiously considered. One point should be borne in mind that the BMD decline only partially accounted for the osteoporotic bone fracture. Literatures indicated that the contribution of the increase in BMD accounted for only 4% of the reduction of vertebral fracture with Rlx compared with 17% with Aln [36–39]. Even though Rlx obtained lower bone mass increment, its adequate risk prevention of vertebral fracture has been well established in MORE studies. In addition, Aln allowed fairly accumulation of microdamage in the vertebra, which would be offset by its increase in bone volume though [40], while the positive effect of Rlx on biomechanical properties might adequately cover the inferior bone mass increment, which ultimately bridge the gap in vertebral fracture prevention between both agents.

Currently, Rlx was infrequently prescribed for women with high risk of nonvertebral fractures [2–4, 6, 8]. In the MORE study, Rlx 60 mg/day did not significant decrease nonvertebral fracture (RR: 0.91 (0.77, 1.07)) compared with placebo [4]. A recent network meta-analysis performed by Murad et al. also demonstrated Aln other than Rlx achieved a significant reduction in nonvertebral fracture compared to placebo (Aln: odds ratio (OR): 0.78 (0.66, 0.92); Rlx: 0.90 (0.76, 1.03)) [6]. But the inferiority of Rlx under Aln in nonvertebral fractures is still highly inconclusive as the definitive difference was not found in RCTs or systematic reviews. A latest database study of over 100,000 postmenopausal women using inverse probability of treatment weights (IPTWs) method for adjustment highlighted that patients treated with either Rlx or Aln had similar rates in nonvertebral fracture after 8 years of adherent treatment [14]. Our pooled data of head-to-head RCTs also questioned the difference of risk reduction in nonvertebral fractures between both agents.

Patients' adherence to drugs, highly influenced by their tolerance, would substantially affect the benefits of drugs [41–43]. Therefore the potential risk of side effects should be thoroughly considered during a decision making. Generally, our review suggested that both drugs were well tolerated with no fatal AEs reported. In particular, Aln increased the incidence of diarrhea while decreased vasomotor events compared to Rlx, which did not require extra medication and seldom caused discontinuation [15]. The increased phlebothrombosis was the main concern for Rlx [44, 45]. However, in our meta-analysis, only 4 venous thrombosis were found (1/990 in Aln, 3/975 in Rlx), which was really rare. Nevetheless, we agreed that Rlx should be contradicted for postmenopausal women who are at high risk of deep vein thrombosis [46]. It was previously demonstrated that postmenopausal women had a greater propensity to adhere to Rlx and higher satisfaction on drug administration compared with Aln mainly due to more GI disorders associated with Aln [47, 48]. In our current analysis, however, the difference of upper GI events and the discontinuation due to AEs between the two agents were balanced. Nevertheless, the greater risk of upper GI disorder of Aln over Rlx was observed when we restricted the analysis to subgroups with daily administration of Aln or subgroups with the age over 65. The results were confirmed by our metaregression analysis. It implied that the daily Aln other than the weekly Aln increased the frequency of GI irritation. Besides, the aged women had more difficulty in taking Aln properly, which contributed to the more GI symptoms [49, 50]. These results provided some references to improve the compliance of Aln. Although there is not any case reported in the included studies due to the short-term followup, the long term risk of atypical fractures and jaw necrosis with Aln treatment should be under careful surveillance.

## 5. Conclusions

Although the moderate-to-high-quality evidence supported that Aln was more effective in increasing the bone mass than Rlx, the moderate-quality evidence suggested no difference in risk prevention of either vertebral or nonvertebral fractures within a followup of 12–24 months. For Aln the weekly strategy would further reduce the upper GI disorders and might gain more bone mass increment compared to the daily treatment. In addition, more diarrhea episodes but less vasomotor events with Aln should also be considered for enhancing the patient compliance during decision making. Which agent, Aln or Rlx, should be preferred for postmenopausal women remained a patient-oriented matter.

## Supplementary Material

Supplementary file 1. Meta-regression analysis of relative risks in the upper gastrointestinal disorders comparison between alendronate and raloxifene [Potential influential factors: A. participants' age; B: daily (1) or weekly (0) administration of alendronate)]
Supplementary file 2. Trim and filled funnel plots of total fracture risk comparison between alendronate and raloxifene.

## Figures and Tables

**Table 1:** Characteristics of the included studies.

Author/year/area	Design	Sample size (Aln/Rlx)	Aln group age (Mean ± SD) BMI (kg/m^2^) BMD (g/cm^2^ or *T*-score)	Rlx group age (Mean ± SD) BMI (kg/m^2^) BMD (g/cm^2^ or *T*-score)	Intervention	Duration (months)	Cofactors	Outcome measurement	Loss to followup (Aln/Rlx)	Industrial funding
Iwamoto et al./2008/Japan [15]	Open-labeled; RCT	122 (61/61)	70.3 (7.6) 21.9 (2.6) LS: 0.620 ± 0.084	68.5 (7.2) 21.7 (2.5) LS: 0.647 ± 0.070	Aln 5 mg/day or Rlx 60 mg/day for 12 months	12	800 mg Ca	(1) Change in BMD in LS measured by DXA at baseline, 6 mo and 12 mo. (2) Changes in serum calcium and phosphorus and bone turnover markers at 1 wk, 6 mo and 12 mo. (3) Changes in the components of the serum lipid profile (4) AEs	18%/14.8% 11/9	None

Johnell et al./2002/Australia^#^ [16]	Multicenter; double blinded; RCT	331 (83/81)	63.7 ± 6.0 24.8 ± 3.8 LS: 0.78 ± 0.14	63.4 ± 6.3 24.8 ± 3.8 LS: 0.77 ± 0.12	Aln 10 mg/day or Rlx 60 mg/day or both for 12 months	12	500 mg Ca plus 400–600 U vit D	(1) Change in BMD in LS, FN measured by DXA at baseline, 6 mo and 12 mo. (2) Change in serum OC, ALP, urinary NTx/Cr at 6 mo and 12 mo. (3) AEs	Total: 17.2%/57	Rlx (Eli Lilly & Co.)

Luckey et al./2004/USA [17]	Multicenter; double blinded; RCT	456 (223/233)	63.8 ± 9.9 25.3 ± 5.2 LS: −2.43 ± 0.78	64.7 ± 9.8 25.3 ± 4.8 LS: −2.5 ± 0.69	Aln 70 mg/week or Rlx 60 mg/day for 12 months	12	500 mg Ca plus 200 U vit D	(1) Change in BMD in TH, LS, and FN measured by DXA at baseline, 6, and 12 mo. (2) Change in serum ALP, urinary NTx/Cr at 6 mo and 12 mo. (3) AEs	19.7%/17.2% 179/173	Aln (Meck & Co.)

Muscoso et al./2004/Italy [18]	Open-labeled; RCT	2000 (1000/100)	71 ± 8 NR NR	64 ± 3 NR NR	Aln 10 mg/day or Rlx 60 mg/day for 24 months	24	1000 mg Ca plus 800 U vit D	(1) Change in BMD in LS measured by DXA at baseline, 12, and 24 mo. (2) Fracture assessment	0	None

Recker et al./2007/USA [19]	Multicenter; double blinded; RCT	1423 (716/707)	65.7 ± 7.8 24.5 ± 4.1 LS: 0.82 ± 0.13 −2.34 ± 1.03	65.5 ± 7.7 24.8 ± 4.2 LS: 0.82 ± 0.13 −2.32 ± 0.99	Aln 10 mg/day or Rlx 60 mg/day for 12 months	24	500 mg Ca plus 400 U vit D	(1) Fracture assessment (2) Change in BMD in TH, LS, and FN measured by DXA at baseline and 24 mo. (3) AEs	2.2%/1.7% 16/12	Rlx (Eli Lilly & Co.)

Sambrook et al./2004/USA [20]	Multicenter; double blinded; RCT	487 (246/241)	61.5 ± 8.2 25.5 ± 3.7 LS: −2.89 ± 0.78	61.8 ± 7.7 25.3 ± 3.9 LS: −2.86 ± 0.76	Aln 70 mg/week or Rlx 60 mg/day for 12 months	12	Ca plus vit D^$^	(1) Change in BMD in LS, FN, TH, and HT measured by DXA at baseline, 6 mo and 12 mo. (2) Change in serum ALP, urinary NTx/Cr at 6 mo and 12 mo. (3) AEs	12%/14% 30/33	Aln (Meck & Co.)

Sanad et al./2011/Egypt [21]	RCT	135 (44/46)	61.7 ± 4.3 25.8 ± 3.1 LS: 0.75 ± 0.05	62.5 ± 3.9 26.5 ± 2.9 LS: 0.73 ± 0.06	Aln 70 mg/week or Rlx 60 mg/day or both for 12 months	12	1500 mg Ca plus 400 U vit D	(1) Change in BMD in TH, LS, and FN measured by DXA at baseline, 6, and 12 mo. (2) Changes in serum ALP, NTx/Cr at 1 wk, 6 mo, and 12 mo. (3) Changes in the components of the serum lipid profile (4) AEs	23.9%/29.5% 13/11	None

Aln: alendronate; Rlx: raloxifene; SD: standard deviation; BMI: body mass index; BMD: bone mineral density; RCT: radomized controlled trials; LS: lumbar spine; mo: months; NR: not reported; Ca: calcium; Vit D: vitamine D; DXA: dual-energy X-ray absorptiometry; TH: total hip; FN: femoral neck; NTx/Cr: urinary cross-linked N-telopeptide of type 1 collagen corrected to creatinine (Cr) excretion; ALP: serum bone-specific alkaline phosphatase; AEs: adverse events.

^#^The fracure data in Johnell's study were not reported in his original article but were cited by Recker's papers and then were extracted and pooled.

^$^The dose of calcium or vitamine D was not clearly stated.

**Table 2:** Methodological quality of eligible randomised controlled trials.

Study	Randomized adequately^a^	Allocation concealed^b^	Blinding^c^	Balance inbaseline	Advoiding selective reporting	Similar cofactors^d^	(%) Loss to followup (Aln/Rlx)^e^	ITT analysis^f^	Quality^g^
Iwamoto et al., 2008 [15]	Unclear	No	No	Yes	Yes	Yes	18%/14.8%	No	Moderate
Johnell et al., 2002 [16]	Adequately	Yes	Double Blinded	Yes	Yes	Yes	total: 17.2%	Yes	High
Luckey et al., 2004 [17]	Adequately	Yes	Double Blinded	Yes	Yes	Yes	19.7%/17.2%	Yes	High
Muscoso et al., 2004 [18]	Unclear	No	No	Yes	Yes	Yes	0	Yes	Moderate
Recker et al., 2007 [19]	Adequately	Yes	Double Blinded	Yes	Yes	Yes	2.2%/1.7%	Yes	High
Sambrook et al., 2004 [20]	Adequately	Yes	Double Blinded	Yes	Yes	Yes	12%/14%	Yes	High
Sanad et al., 2011 [21]	Unclear	No	Unclear	Yes	Yes	Yes	23.9%/29.5%	No	Moderate

^a^The trials could get an “Yes” if their randomization schedules were explicitly described.

^b^Only the trials which mentioned that they concealed the process of patients assignment could get a “Yes.”

^c^The trials were considered as “Double Blinded” if a placebo was adequately adopted to blind both patients and investigators.

^d^In all the included studies, patients in both groups took calcium and vitamine D as supplementations equally.

^e^Less than 20% loss to follow-up rate was considered acceptable.

^f^ITT: intention to treat. Explicit description of the loss to followup was provided in all the included trials, but only which mentioned ITT analysis of the missing data could get a “Yes.”

^g^The frequences of positive responses >5 means “High”; 4 or 5 means “Moderate”; ≦3 means “Low.”

**Table 3:** ^
$^GRADE evidence profile: randomized controlled trials of comparison between Aln and Rlx for postmenopausal women.

No. of trials (No. of women)	Summary of finding	Quality of evidence					
	Magnitude of effect (95% CI)	Risk of bias	Inconsistency	Indirectness	Imprecision	Publication bias	Quality
Efficacy profile							
Antifracture evaluation							
Total fractures risk							
6 trials (3742)	RR: 1.12 (0.75 to 168)	Low	No	Direct	Yes^c^	Unlikely	Moderate
Vertebral fractures risk							
3 trials (2634)	RR: 1.30 (0.66 to 2.54)	Low	No	Direct	Yes^c^	Unlikely	Moderate
Nonvertebral fractures risk							
4 trials (3455)	RR: 0.95 (0.54 to 1.68)	Low	No	Direct	Yes^c^	Unlikely	Moderate
Surrogate anti-fracture evaluation							
LS BMD at 12 months							
6 trials (2396)	WMD: 2.92 (2.23 to 3.62)	Low	Inconsistency^b^	Direct	No	Unlikely	Moderate
5 trials (2274)*	WMD: 2.37 (2.17 to 2.58)	Low	No	Direct	No	Unlikely	High
FN BMD at 12 months							
4 trials (1174)	WMD: 0.84 (0.32, 1.36)	Low	Inconsistency^b^	Direct	No	Unlikely	Moderate
TH BMD at 12 months							
3 trials (1009)	WMD: 1.25 (1.02, 1.49)	Low	Inconsistency^b^	Direct	No	Unlikely	Moderate

Safety profile							
Risk of upper GI events							
6 trials (2708)	RR: 1.10 (0.77 to 1.58)	Low	Inconsistency^b^	No	Yes^c^	Unlikely	Low
5 trials (2221)^#^	RR: 1.30 (1.04 to 1.63)	Low	No	No	No	Unlikely	High
Risk of discontinuations							
5 trials (2642)	RR: 1.03 (0.77 to 1.36)	Low	No	No	Yes^c^	Unlikely	Moderate
Risk of VT events							
3 trials (1934)	RR: 0.52 (0.10 to 2.86)	Low	No	No	Yes^c^	Unlikely	Moderate
Risk of diarrhea events							
3 trials (1600)	RR: 2.33 (1.21 to 4.49)	High^a^	No	No	No	Unlikely	Moderate
Risk of vasomotor events							
2 trials (943)	RR: 0.47 (0.27 to 0.81)	Low	No	No	No	Unlikely	High
Risk of vasodilatation events							
3 trials (1643)	RR: 0.74 (0.54 to 1.01)	Low	No	No	Yes^c^	Unlikely	Moderate

Aln: Alendronate; Rlx: Raloxifene; LS: lumbar spine; FN: femoral neck; TH: total hip; BMD: bone mineral density; WMD: weighted mean differences; RR: risk ratios; CI: confidence interval.

^a^Only 2 or 3 trials are included, of which 2 trials are with high risk of bias; then evidence was rated down.

^b^Statistical heterogeneities (*I* ^2^ > 50%) across studies are detected; therefore quality was decreased.

^c^95% confidence interval included both important superiority and inferiority; then quality was downgraded.

*After excluding Iwamoto's study (the eldest women involved).

^#^After excluding Sambrook's study (the youngest women involved).

^$^GRADE Working Group grades of evidence: high quality: further research very unlikely to change confidence in estimate of effect; moderate quality: further research likely to have important impact on confidence in estimate of effect and may change estimate; low quality: further research very likely to have important impact on confidence in estimate of effect and likely to change estimate; very low quality: very uncertain about estimate.

**Table 4:** WMD of percentage changes from baseline in BMD between Clx.

Studies	6 months			12 months			24 months		
	Aln	Rlx	WMD	Aln	Rlx	WMD	Aln	Rlx	WMD
Lumbar spine	N1	N2	IV, Ran, 95% CI	N1	N2	IV, Ran, 95% CI	N1	N2	IV, Fixed, 95% CI
Iwamoto et al., 2008 [15]	61	61	2.60 [1.93, 3.27]	61	61	5.60 [4.93, 6.27]	/	/	/
Johnell et al., 2002 [16]	83	82	1.40 [0.82, 1.98]	83	82	2.20 [1.62, 2.78]	/	/	/
Luckey et al., 2004 [17]	223	233	1.41 [1.06, 1.76]	223	233	2.50 [2.15, 2.85]	/	/	/
Muscoso et al., 2004 [18]	/	/	/	1000	100	2.20 [2.03, 2.37]	1000	100	4.80 [4.55, 5.05]
Recker et al., 2007 [19]	/	/	/	/	/	/	61	61	2.95 [2.28, 3.62]
Sambrook et al., 2004 [20]	246	241	1.74 [1.40, 2.08]	246	241	2.60 [2.26, 2.94]	/	/	/
Sanad et al., 2011 [21]	31	35	1.16 [0.24, 2.08]	31	35	2.65 [1.73, 3.57]	/	/	/
Total (95% CI)	**644**	**652**	**1.66 [1.28, 2.05]**	**1644**	**752**	**2.92 [2.23, 3.62]**	**1061**	**161**	**3.90 [2.09, 5.71]**
Heterogeneity			*P* < 0.01, *I* ^2^ = 65%			*P* < 0.01, *I* ^2^ = 95%			/

Femoral neck			IV, Fixed, 95% CI			IV, Ran, 95% CI			IV, Fixed, 95% CI
Johnell et al., 2002 [16]	83	82	0.13 [−0.45, 0.71]	83	82	1.00 [0.42, 1.58]	/	/	/
Luckey et al., 2004 [17]	223	233	0.19 [−0.16, 0.54]	223	233	0.30 [−0.05, 0.65]	/	/	/
Recker et al., 2007 [19]	/	/	/	/	/	/	61	61	1.47 [0.80, 2.14]
Sambrook et al., 2004 [20]	246	241	0.63 [0.29, 0.97]	246	241	1.20 [0.86, 1.54]	/	/	/
Sanad et al., 2011 [21]	31	35	0.50 [−0.42, 1.42]	31	35	0.94 [0.02, 1.86]	/	/	/
Total (95% CI)	**583**	**591**	**0.38 [0.16, 0.60]**	**583**	**591**	**0.84 [0.32, 1.36]**	**61**	**61**	**1.47 [0.80, 2.14]**
Heterogeneity			*P* = 0.261, *I* ^2^ = 25%			*P* < 0.01, *I* ^2^ = 78%			/

Total hip			IV, Ran, 95% CI			IV, Fixed, 95% CI			IV, Fixed, 95% CI
Luckey et al., 2004 [17]	223	233	0.75 [0.40, 1.10]	223	233	1.00 [0.65, 1.35]	/	/	/
Recker et al., 2007 [19]	/	/	/	/	/	/	61	61	2.12 [1.45, 2.79]
Sambrook et al., 2004 [20]	246	241	1.28 [0.94, 1.62]	246	241	1.50 [1.16, 1.84]	/	/	/
Sanad et al., 2011 [21]	31	35	0.41 [−0.51, 1.33]	31	35	1.20 [0.28, 2.12]	/	/	/
Total (95% CI)	**500**	**509**	**0.91 [0.44, 1.38]**	**500**	**509**	**1.25 [1.02, 1.49]**	**61**	**61**	**2.12 [1.45, 2.79]**
Heterogeneity			*P* = 0.05, *I* ^2^ = 68%			*P* = 0.13, *I* ^2^ = 51%			/

BMD: bone mineral density; Aln: Alendronate; Rlx: Raloxifene; SD: standard deviation; WMD: weighted mean differences; IV: inverse variance method; Ran: random effects model; CI: confidential interval.

**Table 5:** Subgroup analysis of the main meta-analysis comparing Aln and Rlx.

Factors	Subgroups	Risk of total fracture		LS BMD at 12 months		LS BMD at 12 months (Absence of outlier)		Risk of upper GI disorders		Risk of upper GI disorders (Absence of outlier)	
		*N *	RR (95% CI)	*N *	WMD (95% CI)	*N *	WMD (95% CI)	*N *	RR (95% CI)	*N *	RR (95% CI)
	Total	6	1.19 [0.78, 1.83]	6	2.92 [2.23, 3.62]	5	2.37 [2.17, 2.58]	6	1.10 [0.77, 1.58]	5	**1.30 [1.04, 1.63]**
	Heterogeneity		*P* = 0.78; *I* ^2^ = 0%		***P* < 0.01; *I^2^* = 95%**		*P* = 0.19; *I* ^2^ = 35%		***P* = 0.07; *I^2^* = 52%**		*P* = 0.83; *I* ^2^ = 0%
Patterns of treatments in Aln	Daily	4	1.23 [0.77, 1.98]	3	2.39 [2.23, 2.56]	2	2.20 [2.03, 2.37]	3	**1.34 [1.04, 1.72]**	3	**1.34 [1.04, 1.72]**
	Weekly	2	0.86 [0.39, 1.90]	3	2.56 [2.32, 2.79]	3	2.56 [2.32, 2.79]	3	0.92 [0.49, 1.72]	2	1.21 [0.67, 2.20]
			*P* = 0.44		*P* = 0.26		***P* = 0.01**		*P* = 0.27		*P* = 0.76

Age	≥65	3	1.17 [0.72, 1.91]	2	3.88 [0.55, 7.22]	1	/	2	**1.32 [1.01, 1.73]**	2	**1.32 [1.01, 1.73]**
	<65	3	1.01 [0.48, 2.14]	4	2.51 [2.29, 2.73]	4	/	4	1.03 [0.61, 1.74]	3	1.26 [0.83, 1.90]
			*P* = 0.74		*P* = 0.42		/		*P* = 0.41		*P* = 0.85

Methodological quality^a^	Low	2	1.39 [0.59, 3.27]	3	3.48 [1.26, 5.69]	2	2.22 [2.04, 2.39]	2	1.67 [0.67, 4.13]	2	1.67 [0.67, 4.13]
	High	4	1.05 [0.66, 1.67]	3	2.50 [2.28, 2.72]	3	2.50 [2.28, 2.72]	4	1.03 [0.68, 1.57]	3	1.28 [1.01, 1.62]
			*P* = 0.57		*P* = 0.39		*P* = 0.05		*P* = 0.35		*P* = 0.58

Sample size^b^	≥400	5	/	3	2.40 [2.13, 2.67]	3	2.40 [2.13, 2.67]	3	0.95 [0.57, 1.58]	2	1.26 [0.99, 1.61]
	<400	1	/	3	3.49 [1.25, 5.73]	2	2.33 [1.84, 2.82]	3	1.55 [0.88, 2.77]	3	1.55 [0.88, 2.76]
			/		*P* = 0.34		*P* = 0.80		*P* = 0.21		*P* = 0.51

Funding	Aln	2	0.86 [0.39, 1.90]	2	2.55 [2.31, 2.79]	2	/	2	0.77 [0.43, 1.37]	1	/
	Rlx	2	1.17 [0.66, 2.07]	2	2.20 [1.62, 2.78]	1	/	2	**1.34 [1.04, 1.74]**	2	/
	None	2	1.39 [0.59, 3.27]	3	3.48 [1.26, 5.69]	3	/	2	1.67 [0.67, 4.13]	2	/
			*P* = 0.70		*P* = 0.38		/		*P* = 0.18		/

Aln: Alendronate; Rlx: Raloxifene; *N*: number of trials; WMD: weighted mean differences; CI: confidential interval; RR: risk ratios; LS: lumbar spine; BMD: bone mineral density; ALP: serum bone-specific alkaline phosphatase; AEs: adverse effects; GI: gastrointestinal.

^a^Results were not changed when subgroups were divided by adequate randomization, concealing allocation, blinding, applying ITT analysis.

^b^The cutoff of sample size was defined according to a threshold rule-of-thumb.

Bold font means the statistic significances were existed.
