# Supplementary figures and images for: Alendronate versus Raloxifene for Postmenopausal Women: A Meta-Analysis of Seven Head-to-Head Randomized Controlled Trials
Source: Int J Endocrinol. 2014 Jan 5;2014:796510. doi: 10.1155/2014/796510 (PMC3912893; doi:10.1155/2014/796510)

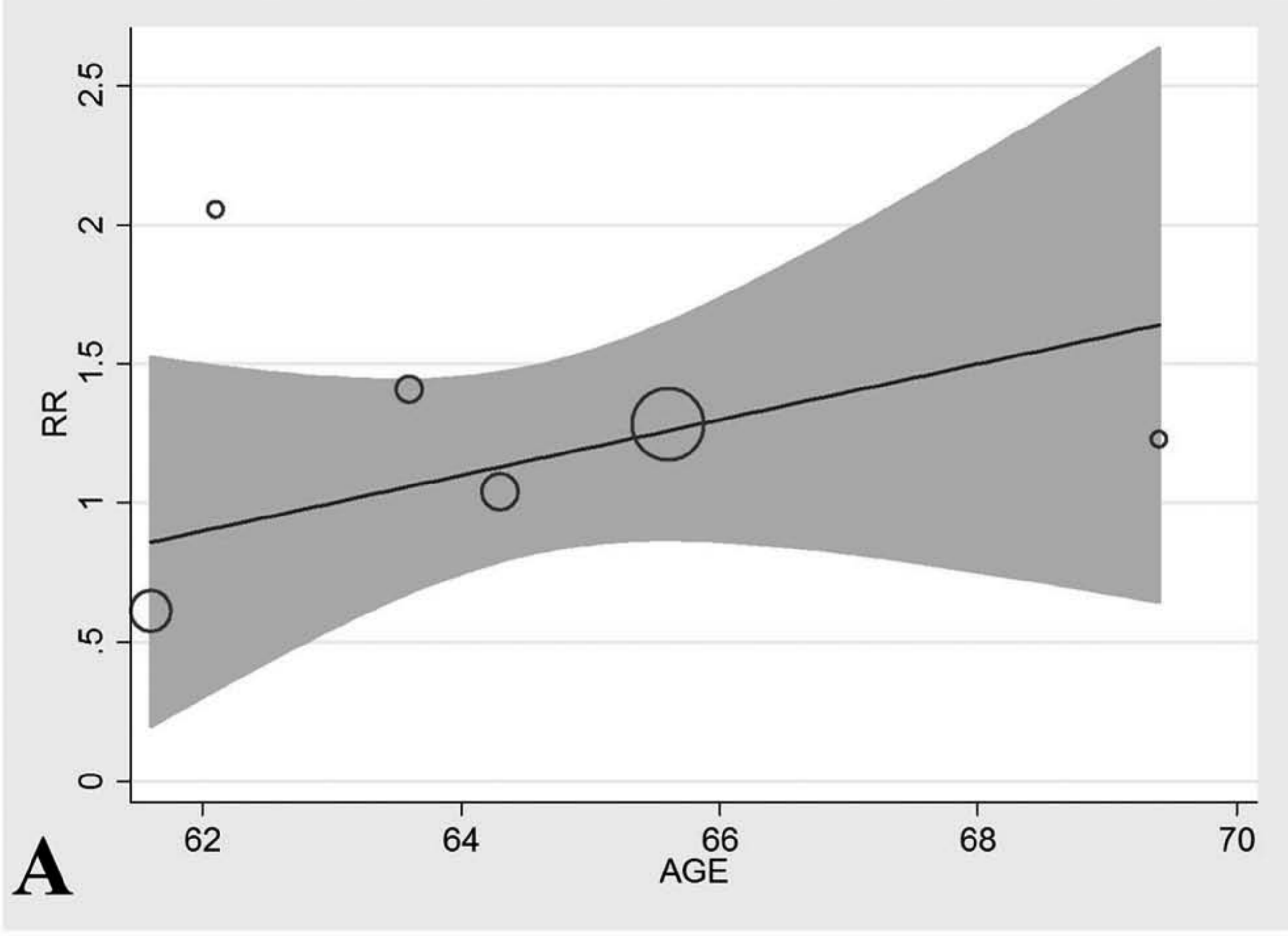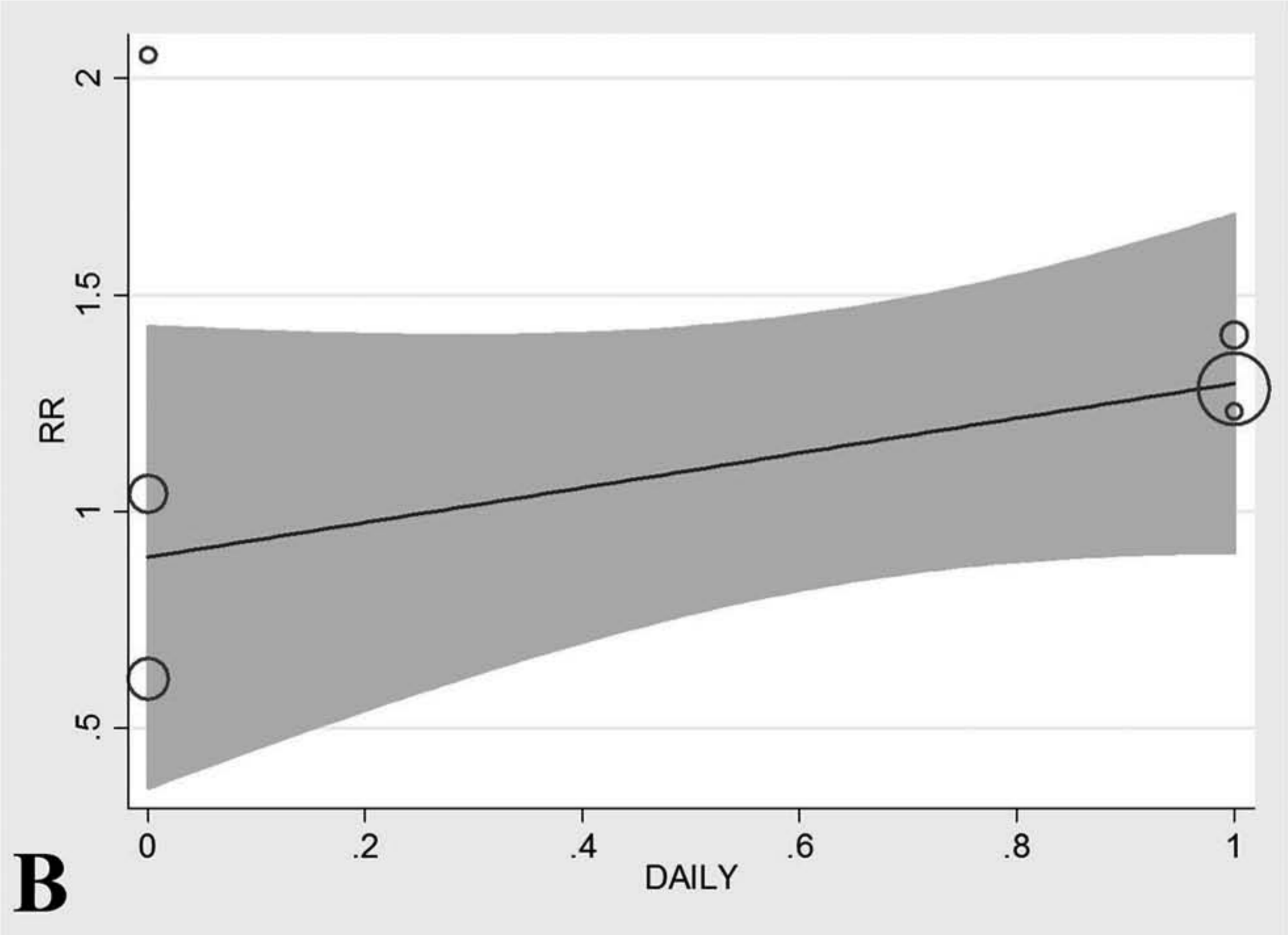

Supplementary file 1

Filled funnel plot with pseudo 95% confidence limits

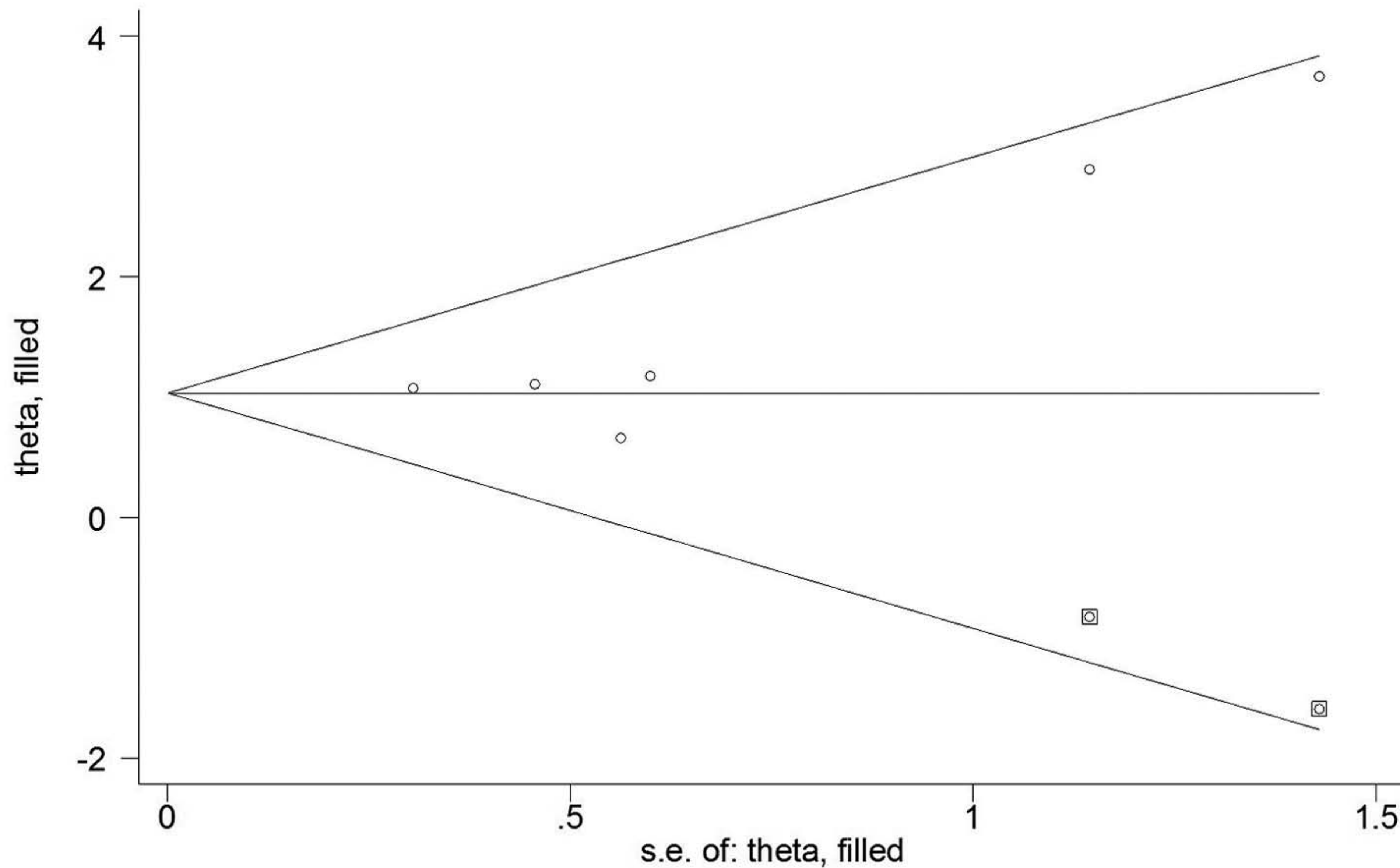

Supplement: Supplementary file 1 — Meta-regression analysis of relative risks in the upper gastrointestinal disorders comparison between alendronate and raloxifene [Potential influential factors: A. participants' age; B: daily (1) or weekly (0) administration of alendronate)] Supplementary file 2. Trim and filled funnel plots of total fracture risk comparison between alendronate and raloxifene. [file 796510.f1.pdf]
